# Exposed Nerves

**Published:** 1860-01

**Authors:** G. Ezra M’Kown


### EXPOSED NERVES.

BY G. EZRA M'KOWN.

THE disease indicated in the above caption is one *deserving* the consideration of every practitioner of dentistry; and having been interested by the remarks in the DENTAL COSMOS, between one of your able corps and Prof. Taft, of Cincinnati, not wishing either pro. or con. to enter into the effects of "Nitric Acid" as applied to "recently exposed nerves," but with *your permission*, I wish to lay before the readers of the DENTAL COSMOS the method I have employed for the last three years, and suggested to me by Dr. Amber, of Cleveland, during an operation on my teeth. A case to illustrate. In excavating a cavity in the posterior proximal surface of the left superior lateral incisor, it became evident that the nerve was covered by a very thin lamina of bone, and in excavating to form the proper shape to retain the filling, this part also became loose, and was removed, causing the pulp to bleed *profusely*. Applied a pledget of cotton, saturated with creosote and tannic acid, to arrest the hæmorrhage; retained it from three to five minutes; the pulp was found cauterized and of a whitish color. The cavity was then thoroughly washed with tepid water and camphor, and after being properly dried, a small parcel of cotton, saturated with ol. creosote, was put in contact with the pulp as an *intervening* substance. The cavity of decay was filled with "crystal gold," introduced in such a manner as to protect that part from the *main* or *direct* pressure. Two years and a half have elapsed, and it has never given any trouble, neither has it lost its natural color or vitality. One more case (out of *many* I *could mention.*) October 1, 1857.—Mr. B., aged 24 years, nervous temperament, had a number of teeth filled. The first right superior molar had a large cavity in the anterior proximal surface; dentine sensitive; excavated without wounding the pulp; cauterized with creosote, and filled with "Hill's stopping." July 6, 1859.—The tooth having caused no inconvenience, removed the stopping to insert a more permanent filling; to my surprise, and the gratification of my patient, we found the dentine not only in a normal condition, but the nerve protected by a thick stratum of dentine, resembling crusta petrosa in appearance, but not in structure. To be brief—it was excavated and filled without any inconvenience. The tooth and contiguous parts have continued in a normal condition thus far. Now I do not advocate *this* as anything new, but merely as my *mode* of *practice;* and, taking teeth as they are presented, if the nerves *have* been previously exposed, with no symptoms of periostitis, have had a better percentage of success than by destroying and extirpating the nerve, and filling the *fang*, or by any other method (that has come under my observation) that has been devised.—*Dental Cosmos.*

